# Extracellular Vesicles and Chemotherapy Resistance in the AML Microenvironment

**DOI:** 10.3389/fonc.2020.00090

**Published:** 2020-02-14

**Authors:** Jill Nehrbas, John T. Butler, Ding-Wen Chen, Peter Kurre

**Affiliations:** ^1^Comprehensive Bone Marrow Failure Center, Children's Hospital of Philadelphia, Philadelphia, PA, United States; ^2^Abramson Cancer Center, Perelman School of Medicine, University of Pennsylvania, Philadelphia, PA, United States; ^3^Department of Biomedical Engineering, Oregon Health & Science University, Portland, OR, United States; ^4^Department of Pediatrics, Oregon Health & Science University, Portland, OR, United States

**Keywords:** acute myeloid leukemia, extracellular vesicles, chemoresistance, bone marrow microenvironment, stroma

## Abstract

Extracellular vesicle (EV) trafficking provides for a constitutive mode of cell-cell communication within tissues and between organ systems. Different EV subtypes have been identified that transfer regulatory molecules between cells, influencing gene expression, and altering cellular phenotypes. Evidence from a range of studies suggests that EV trafficking enhances cell survival and resistance to chemotherapy in solid tumors. In acute myeloid leukemia (AML), EVs contribute to the dynamic crosstalk between AML cells, hematopoietic elements and stromal cells and promote adaptation of compartmental bone marrow (BM) function through transport of protein, RNA, and DNA. Careful analysis of leukemia cell EV content and phenotypic outcomes provide evidence that vesicles are implicated in transferring several known key mediators of chemoresistance, including miR-155, IL-8, and BMP-2. Here, we review the current understanding of how EVs exert their influence in the AML niche, and identify research opportunities to improve outcomes for relapsed or refractory AML patients.

## Introduction

Acute myeloid leukemia (AML) is a genetically heterogenous disease that arises from abnormal proliferation of hematopoietic stem cells (HSCs) (1). Although most patients respond to current treatment strategies, a majority of patients will ultimately experience relapse (2). Patient survival rates remain low due largely to high rates of relapse as a consequence of intrinsic and extrinsic resistance (3). Survival of residual leukemic cells that give rise to relapse is typically attributed to clonal genetic adaptations that may precede treatment or emerge during chemotherapy (4). Extrinsic mechanisms that actively confer protection of leukemic cells from complete elimination in the bone marrow (BM) following therapy are increasingly recognized for their role in chemo-resistance and clonal persistence; examples include nuclear factor kappa-light-chain-enhancer of activated B-cells (NF-kB) mutation, drug efflux pump activation, and adaptive action of microRNA (miRNA) mediated cell-cell crosstalk mechanisms (5, 6).

Extracellular vesicles (EVs) are membrane-bound particles secreted from cells, carrying a variety of nucleic acid and protein cargo active in cell-cell communication (7). As a constitutive cellular mechanism for the transport of bioactive cargo, EVs promote therapy resistance in the bone marrow niche (8, 9). Now EV content and functional analyses are beginning to shed light on the some key mediators involved in chemoresistance such as miRNA-155 (miR-155), interleukin 8 (IL-8), and bone morphogenic protein 2 (BMP-2) (10–12). In this review, we critically examine the current evidence connecting EV trafficking and resistance to both chemo- and immunotherapies, while highlighting key areas of research.

## Acute Myeloid Leukemia

AML is a genetically heterogeneous disease characterized by the successive acquisition of mutations in HSCs that cause unchecked proliferation and a coincident differentiation arrest (1). At diagnosis, patients typically present with symptoms arising from leukocytosis, anemia, and thrombocytopenia. At an annual incidence of 4.3 per 100,000 persons, AML is the most common acute leukemia in adults, and second to acute lymphoid leukemia (ALL) in children (13). The overall 5-year survival rate is <50% in young adults, which drastically decreases in elderly patients with a 2-year survival rate of <20% (3). With well over 200 identified molecular lesions illustrating overall disease heterogeneity, some common recurring cytogenetic abnormalities are seen, including translocations between chromosomes 8 and 21, deletions in chromosomes 5 and 7, and inversions in chromosome 16 (3). Likewise, activating mutations in several oncogenes have been shown to alter key components in cell cycle regulation such as tumor protein p53, fms-like tyrosine kinase 3 (FLT-3), WNT, and MYC (3). Individually and in combination, these genetic abnormalities carry important information for both diagnostic and treatment stratification of AML. For example, depending on genetic context, mutations in nucleophosmin (NPM1) found in about 30% of adult AML cases can denote more favorable outcomes for patients (14). Meanwhile, patients with FLT-3 internal tandem duplication (FLT-3 ITD) account for ~25% of AML cases and generally denote a poor prognosis, except in combination with NPM1 (15). Other oncogene mutations such as mutations in the RAS family of proteins, accounting for around 15% of AML cases, are associated with a mixed prognostic impact (16). In spite of the genetic heterogeneity, current AML treatment typically relies on a backbone of cytarabine and anthracyclines in use since the 1970s, with the more recent addition of hypomethylating agents for patients over the age of 65 (17). Critically, whereas most patients initially respond to these treatments, many experience relapse or develop refractory disease (18). While HSC transplant can be a therapeutic option, the overall survival remains between 20 and 60%, due to ineffective salvage treatment regimens (17, 19). Clearly, a better understanding of the barriers to elimination of residual AML cells from their sanctuary in the BM holds untapped therapeutic opportunities for patients.

## Genetic and Functional Adaptation Toward Cell-Autonomous Chemoresistance in AML Cells

Development of drug resistance during chemotherapy is a primary challenge to sustaining AML remission in patients. This can occur as a direct consequence of mutations, or indirectly through signaling pathways or enzyme activities that lead to cancer cell protection (6). Conceptually, clonal evolution under therapy can lead to the emergence of more chemo-resistant clones, that further fuel cell growth and boost the survival advantage (4, 20). For example, some patients acquire FLT-3 ITD mutations during the course of treatment (21, 22). Interestingly, the observation that treatment through tyrosine kinase inhibition efficiently clears peripherally circulating blasts, but not those in the in the BM does not reflect intrinsic genetic events (23).

Non-genetic chemoresistance mechanisms such as p-glycoprotein (P-gp) overexpression, an ATPase efflux pump that export drugs or their active metabolites, are also correlated with poor disease outcomes (24). These direct effects are compounded by the downstream activation of NF-κB signaling that controls cell proliferation and counters apoptosis (6). Mechanistically, NF-κB overactivation protects AML blasts from apoptosis as a result of increased expression of pro-survival BCL-2 proteins (25). Another common mechanism of chemoresistance involves glutathione s-transferase (GST) overexpression, an enzyme that typically protects against reactive electrophiles and DNA damage (26). When overexpressed in cancer, GST has been shown to increase chemoresistance, possibly by catalyzing the binding of glutathione to chemotherapy drugs to minimize their effects, preventing drugs from attacking DNA, or deactivating cisplatin, a common platinum-based component of many chemotherapies that targets DNA in cancer cells (6, 27, 28).

Evidence has also shown that cellular microRNAs are highly involved in the development of drug resistance in AML (29). For example, CXCR4-mediated signaling has been shown to cause chemoresistance in AML cells by downregulating miR-let-7a, which increases transcriptional activation of MYC oncogene and BCL-XL in AML cells (30). Several miRNAs that bind to DNA damage regulatory proteins are overexpressed in AML. For example, overexpression of miR-181a in AML cells downregulates ATM, a critical checkpoint kinase required for cell cycle arrest, and in turn promotes cancer cell growth (31). miR-128 is also overexpressed in AML, which downregulates RAD51 and reduces DNA damage response (32). Downregulation in a number of different miRNAs such as miRs−15a (33),−15b (34),−125b-5p (35),−139-5p (36),−145 (37), and−181a (38) have been reported in other forms of cancer, which collectively suggests that elevated BCL2 translation plays a major role in resistance development. Intriguingly, EV secretion may alter leukemogenic properties in a cell autonomous fashion and promotes expansion and persistence of leukemia initiating cells (39).

## AML Niche Conversion and Acquisition of Non-cell-autonomous Chemoresistance

**Figure 1:**
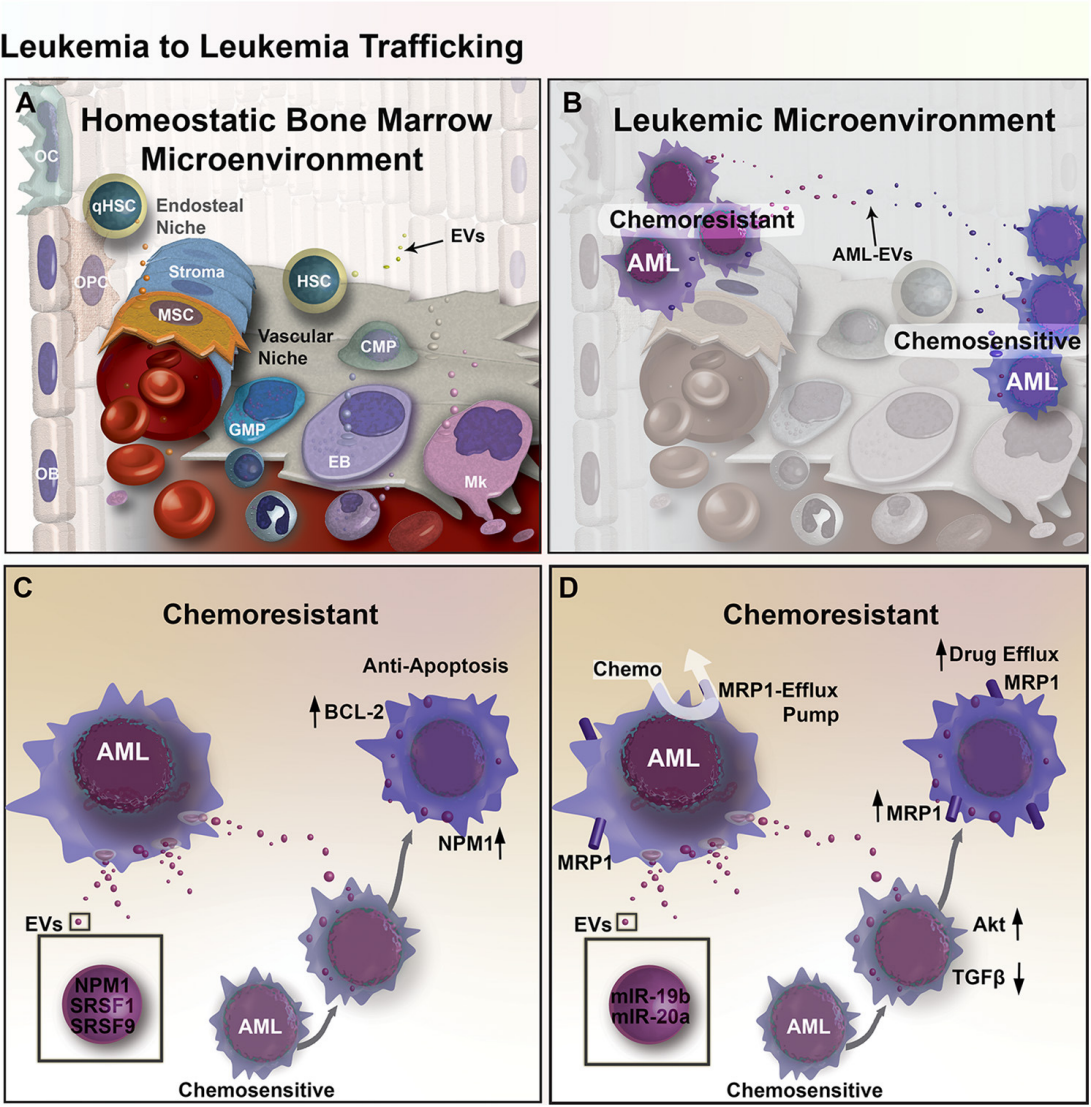
EV mediated transfer of chemoresistance between leukemia cells in the BM microenvironment. **(A)** Diagram of the BM microenvironment, composed of the hematopoietic niche (right) stromal compartment (left). Hematopoietic Stem and Progenitor Cell (HSPC) give rise to Common Myeloid Progenitors (CMP), Granulo-Monocytic Progenitor Cells (GMP), Erythroblasts (EB), Megakaryocytes (Mk), and many other cell types that populate the cells of the blood. In the stromal compartment, Mesenchymal Stromal Cells (MSC) give rise to Osteoprogenitor Cells (OPC) and Osteoblasts (OB), together these cells function to form bone and regulate hematopoiesis in part through EV-mediated signaling. **(B)** Expansion of leukemic cells results in microenvironmental dysregulation. EV trafficking between AML cells transfers regulatory factors that induce resistance to chemotherapy. **(C)** Chemo-experienced AML cells shed EVs containing NMP1, SRSF1, and SRSF9, which increase apoptosis resistance through upregulation of BCL-2 and NPM1 in unexperienced recipient AML cells. **(D)** EVs from chemo-experienced AML cells also contain miR-19b and−20a, which reduce TGF-β signaling and increase Akt signaling and the expression of MRP1 chemo-efflux pump in recipient AML cells.

The bone marrow compartment comprises a range of cell types that form HSC supportive microenvironments: endosteal (comprised of osteoblasts, osteocytes, and osteoclasts) and vascular (comprised of endothelial cells and megakaryocytes) niches (Figure 1A). The endosteal niche in the internal surface of the bone helps maintain stem cell quiescence through the binding of osteoblasts to HSCs (40), while the vascular niche promotes the proliferation and differentiation of HSCs through crosstalk between endothelial cells and HSCs (40). Through successive adaptation of these niches, the BM microenvironment contributes to leukemia cell proliferation and survival (Figure 1B) (41–43). In the endosteal niche, AML cells may induce mesenchymal stromal cells (MSCs) toward an accelerated osteoblastic differentiation where accumulation of these immature osteoblasts can lead to an overall decrease in the number of functional osteoblasts (5). One study showed that AML and myelodysplastic syndrome patients had an osteoblast count 55% lower than healthy controls, consistent with observations in mice bearing acute leukemia, that exhibited osteoblast deficits inversely proportional to circulating blast burden and survival (44).

Emerging evidence also suggests that AML blasts localize and integrate with the BM vascular niche. A study by Cogle et al. showed that some AML cells have the capacity to fuse with vascular endothelium (vascular-AML), acquiring several endothelial characteristics before assuming a state of quiescence (45). Moreover, these vascular-AML cells remain leukemogenic and were able to give rise to leukemia following transplantation into healthy mice, suggesting that AML cells that have integrated into the vascular niche may be implicated in relapse (45). Within the BM microenvironment, crosstalk between AML cells and BM stromal cells (BMSCs) also plays a role in chemoresistance development. For example, AML cells can induce secretion of growth arrest-specific gene 6 (GAS6) in BMSCs, which protects AML cells from cell death (46). More recently, findings further illustrate the significance of BMSC-AML interactions, whereby AML cells remodel the endosteal vascular niche by producing pro-inflammatory and anti-angiogenic cytokines that results in the loss of endosteal blood vessels and BMSCs surrounding blood vessels and bones (47). These endosteal AML cells were found to have elevated expression of tumor necrosis factor (TNF) and CXCL2, which are involved in vascular destruction and angiogenesis inhibition, respectively. In addition, AML-induced remodeling of the niche disrupts HSC homing, and reduces the efficiency of drug transport, increasing the risk of chemoresistance (48).

## Mechanisms of Cell-Cell Crosstalk

Several mechanisms of intercellular communication contribute to the conversion of BM from a homeostatic-supportive compartment to an oncogenic compartment (Table 1). Traditionally, cells communicate via soluble factors such as cytokines, neurotransmitters, hormones, and growth factors, where they act in a juxtacrine, paracrine, or endocrine manner. In recent years, EVs have emerged as a novel mechanism of intercellular communication. Based on their method of biogenesis, content, and physical properties, EVs can be broadly categorized into exosomes, exomeres, microvesicles, and apoptotic bodies (52, 53). Like other types of EVs, exosomes sized at up to 120 nm, are equipped to traffic proteins, lipids, mRNAs, multiple types of non-coding RNAs, DNAs, and chemokines/cytokines between cells (52). Observations in multiple types of cancer (Table 2) indicate that cells with malignant potential release exosomes at an increased rate, selectively capturing certain molecules that promote the proliferation of cancer cells, metastasis, and drug resistance (59). Exosomes have been implicated in the transformation of BM microenvironment into a leukemia-permissive space. For example, one recent xenograft study revealed that AML-derived exosomes upregulate DKK1 in BMSCs, suppressing hematopoiesis and inhibiting osteoblast differentiation via Wnt pathway signals (43). Specifically, these AML derived exosomes downregulate gene expression that otherwise sustains normal hematopoiesis (*Igf1, Cxcl12, Kitl*, and *Il-7*), and genes involved in normal bone development (*Ocn* and *Col1A1*). In addition to playing a key role in transforming the BM niche, exosomes may in part contribute toward the protection of leukemia blasts during chemotherapy treatment (43).

**Table 1:** Modes of cell-cell communication and their potential role in drug resistance.

**Mode of communication**	**Action**	**Effects in cancer**	**Representative source**
Exosomes	Membrane-bound vesicles that transport molecules between cells	Transfer proteins, miRNA, and other molecules that increase proliferation, metastasis, and chemoresistance	(29)
Cytokines	Small proteins that are secreted from cells and act on receptors in order to have effects on immunity, inflammation, and hematopoiesis	Alterations in levels of pro- and anti-inflammatory cytokines has been shown to increase cell proliferation, survival, and resistance to chemotherapy	(49)
Gap junctions	Integral membrane proteins that enable a transfer of ions and small molecules that act as second messengers	Gap junctions have been shown to be involved in cell communication in leukemia and chemosensitivity	(50)
Tunneling nanotubes	Cell surface protrusions that enable exchange of signals, proteins, pathogens, and organelles	Have been shown to transfer mitochondria, resulting in increased drug resistance	(51)

**Table 2:** Transfer of molecular cargo via exosomes in cancer.

**Cell type**	**EV cargo**	**Example**	**Effect**	**Representative source**
AML	mRNA	IGF-IR	Upregulate VEGF expression and modulate proliferative signaling	(8)
Breast cancer	miRNA	miR-155	Involved in TGF-β-induced epithelial-mesenchymal transition, invasion, metastasis, and drug resistance	(54)
Non-small cell Lung cancer	Long non-coding RNA	MALAT-1	Promotes tumor growth and migration and prevents apoptosis in tumor cells	(55)
Gastric cancer	siRNA	HGF	Downregulates HGF expression, inhibits tumor growth, and angiogenesis, suppresses proliferation and migration of vascular and cancer cells	(56)
AML	Proteins	BMP-2	Contributes to an osteogenic differentiation bias and plays a role in the induction of the unfolded protein response	(12)
Pancreatic cancer	DNA	DNA spanning all chromosomes with mutations in KRAS and P53	Regulatory influence on recipient cells	(57)
AML	Cytokines	TGF-β1	Down-regulate NKG2D expression and impair natural killer cell cytotoxicity	(58)

Another well-characterized mechanism of cell-cell crosstalk is the transmission of signals involving direct cell-cell contacts such as gap junctions composed of membrane protein called connexins, that transfer ions and small molecules serving as second messengers in a neighboring cell. Connexins have been shown to act as either tumor promoters or suppressors in breast cancer, prostate cancer, and brain cancer (60–63). A more recently described method of direct cell-cell communication is through tunneling nanotubes (TNTs), cell membrane protrusions composed of F-actin that transiently connect cells to exchange proteins, pathogens, or even organelles. Formation of TNTs appears to increase in cells under stress, potentially increasing survival through the exchange of mitochondria (64, 65). TNT trafficking of bioactive cargo influences differentiation, metabolism and immune response (65).

## Directional Transfer of Chemoresistance Via EVs Between AML Cells

An elegant study in glioma reported by Al-Nedawi provided the first compelling evidence that shows EV-mediated intratumoral transfer of functional resistance factors between differentially chemo-sensitive cancer cells (66). In recent years, emerging evidence has shown that EV-mediated resistance transfer also exists in AML (Figure 1B), where proteins and miRNAs can be transported by EVs between AML cells and elicit differential gene expression profiles and cell activity in recipient AML cells (67, 68). More specifically, a co-culture experiment showed that chemo-resistant AML cells can induce chemoresistance in chemo-sensitive AML cells by triggering anti-apoptotic protein BCL-2 upregulation in chemo-sensitive AML cells, while chemo-sensitive AML cells could not induce BCL-2 upregulation in other chemo-sensitive AML cells (67). Aberrant expression of apoptosis-regulating proteins may allow AML cells to escape apoptosis, which can also be used to predict minimal residual disease (69). In addition to anti-apoptotic effects, upregulation of BCL-2 family of proteins also have implications in reducing unfolded protein response (UPR) stress in cancer cells through IRE1α pro-survival pathway activation (67). Activation of the IRE1α pathway involves the splicing of Xbp1, leading to endoplasmic reticulum (ER) chaperone synthesis and ER-associated protein degradation complex formation that ultimately supports cancer cells in adapting to ER-stress (70). Proteomic analysis of secretomes derived from both apoptotic-resistant and apoptosis-sensitive AML cells reveal an increase of apoptosis-regulating proteins are present in the secretome of apoptosis-sensitive AML cells (67). Furthermore, functional clusters of proteins within apoptosis-resistant cell-derived exosomes were associated with gene ontology (GO) terms such as non-coding RNA (ncRNA) metabolism, DNA replication and repair, translation elongation, and mRNA splicing. In addition, these exosomes contained NPM1, a protein associated with increased apoptosis-resistance. Meanwhile, exosomes from apoptosis-sensitive cells were more strongly associated with inflammatory and stress response GO terms. These findings suggest that apoptosis resistance can be transferred from apoptosis-resistant to apoptosis-sensitive AML cells via exosomes (Figure 1C) (67).

EV-mediated transfer of miRNAs is known to increase chemoresistance in several types of cancer (68, 71, 72). Exosomes derived from BMSCs of AML patients express different miRNA profile compared to healthy controls (10). More specifically, analysis of BMSC-derived exosomes from eight AML patients revealed that miR-155 and miR-375 are consistently enriched in exosomes, unlike exosomes derived from healthy donor BMSCs. Furthermore, the effect of these miRNAs in chemoresistance was demonstrated in a tyrosine kinase inhibitor AC220 challenge, where Molm14 AML cells pretreated with AML BMSC-derived exosomes gained resistance to tyrosine kinase inhibitor and conferred AML cell protection (10).

EV-mediated resistance transfer was also demonstrated in a separate study, where multidrug-resistant AML cells were able to transfer chemoresistance properties to chemo-sensitive AML cells via EV and induce the expression of drug efflux pump multidrug resistance protein 1 (MRP-1) (68). Tracking of fluorescently-labeled EVs derived from chemo-resistant cells showed that those EVs were uptaken by 85% of chemo-sensitive AML cells, which provided increased resistance to daunorubicin. Exposing EV-treated chemo-sensitive AML cells with MRP-1 inhibitor led to increased intracellular retention of daunorubicin following daunorubicin treatment. However, it is unclear whether the exosomes directly transferred MRP-1 or MRP-1 regulatory factors. To better understand the role of exosomes in this gain of chemoresistance, a miRNA analysis of exosomes was performed and showed that exosomes from chemo-resistant cells, in comparison to exosomes from chemo-sensitive cells, contained four-fold more miR-19b and miR-20a, which are often overexpressed in cancer. Both miRNAs play a role in the inhibition of TGF-β signaling (73). Additionally, research suggests that elevated levels of both miRNAs may activate PI3 kinase/Akt signaling, which could lead to the overexpression of MRP-1, supporting the possibility that exosomes mediate the transfer of chemoresistance via miRNAs transport (Figure 1D) (68, 74).

## Directional Transfer Via EVs Between AML and Stromal Elements

**Figure 2:**
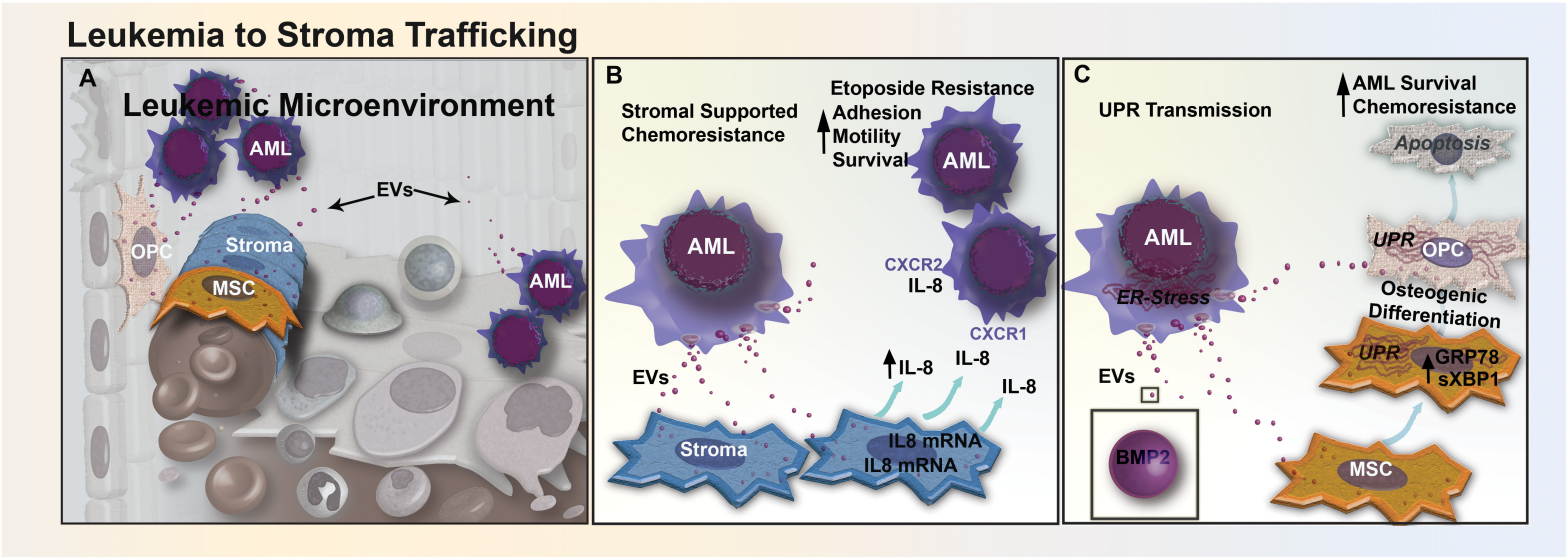
EV trafficking between leukemia and stromal cells reduces sensitivity to chemotherapeutics. **(A)** Bidirectional trafficking between leukemia and stromal cells leads to alterations in both the cellular composition and secretome of the stromal compartment. **(B)** In CML, EV-mediated signaling upregulates the secretion of IL-8 from BM stromal cells. Increased IL-8 in the leukemic microenvironment increases CML cell adhesion, motility, and survival through CXCR1 and CXCR2 engagement, and promotes resistance to etoposide. **(C)** AML cells from the nutrient-depleted leukemic microenvironment exhibit marked endoplasmic reticulum (ER) stress and upregulation of BMP-2. AML-EVs transfer BMP-2 and ER-stress to stromal MSCs and OPCs, activating the unfolded protein response pathway (UPR). UPR activation induces osteogenic differentiation in MSCs and causes increased apoptosis in osteoprogenitor cells, altering the cellular composition of the BM, AML survival, and response to chemotherapy.

In addition to exosome-mediated transfer of chemoresistance phenotypes between chemo-resistant and chemo-sensitive leukemic cells, exosomes can also transport cargo from AML cells to BMSCs that may contribute to the formation of a leukemia-supportive BM microenvironment (Figure 2A). A recent study reported that treatment of AML cells with BMSC-derived exosomes modestly decreased etoposide-induced apoptosis (11). However, in AML cells and BMSCs co-culture, levels of pro-inflammatory cytokine IL-8 significantly increased in BMSCs and protected AML cells from etoposide-induced apoptosis (Figure 2B). Furthermore, inhibition of exosome secretion using an inhibitor of the EV budding regulator neutral sphingomyelinase (GW4869) resulted in a reduction of IL-8 secretion from BMSCs in a BMSC-AML co-culture setting, which had no effect on IL-8 secretion levels in BMSCs cultured alone (75). This finding provides evidence that AML cells secrete exosomes that induce BMSCs to release IL-8, which in turn makes AML cells more resistant to etoposide (11). Similarly, in a study of chronic myelogenous leukemia (CML), IL-8 secreted from BMSCs treated with CML exosomes binds to two transmembrane domain receptors, CXCR-1 and CXCR-2, and increased cell adhesion, motility, and survival of CML cells (76).

Our own recent study suggests that AML exosomes may also modulate the microenvironment by transfer of ER stress responses via exosomes in BMSCs (12). During chemotherapy, leukemic cells can become deprived of oxygen, nutrients, and intracellular calcium, leading to the accumulation of unfolded proteins within the ER (77). This leads to activation of the UPR, decreasing the synthesis of new proteins, increasing unfolded protein degradation and protein folding chaperone levels, while helping cancer cells evade chemo-induced apoptosis (77). We have shown that the transfer of EVs from AML cells to BMSCs can enhance extrinsic chemoresistance and propose that AML exosomes trigger UPR by transferring BMP-2, a protein known to be upregulated in AML (Figure 2C). The trafficking of BMP-2 may cause increased osteogenic differentiation in MSCs, and an increase AML growth by inducing connective tissue growth factor (CTGF) expression in MSCs (42). In our study, ELISA analysis of EVs released from Molm-14 cells treated with Thapsigargin, a drug that induces UPR by inhibiting sarcoplasmic/endoplasmic reticulum Ca2+-ATPase, showed higher levels of BMP-2 than EVs released from untreated Molm-14 cells (12). Additional evidence supports that AML EVs traffic to the ER of MSCs and OPCs *in vitro* and induce upregulation of both GRP78, a key chaperone protein involved in UPR, and spliced Xbp1, a transcription factor for chaperones and ER stress sensors (12). Finally, high levels of BMP signaling have been linked to elevated expression of anti-apoptotic genes (42). Mechanistically, BMP action may involve additional cellular targets, as have been identified in CML where BMP-2 and BMP-4 were found to promote overexpression of the BMPR1a and altered downstream signaling in leukemic stem cells (78). Therapeutically, BMP-mediated leukemic myeloid progenitor expansion can be rescued through neutralization of circulating BMP-2 and BMP-4 proteins using soluble BMP receptor acting as a decoy. Taken together, these observations suggest that BMP-2 trafficked by exosomes influences recipient cell ER stress responses, increasing AML cell survival by altering gene expression and driving osteogenic MSC differentiation.

## Exosomes Protect Leukemia Cells Against Immunotherapy

**Figure 3:**
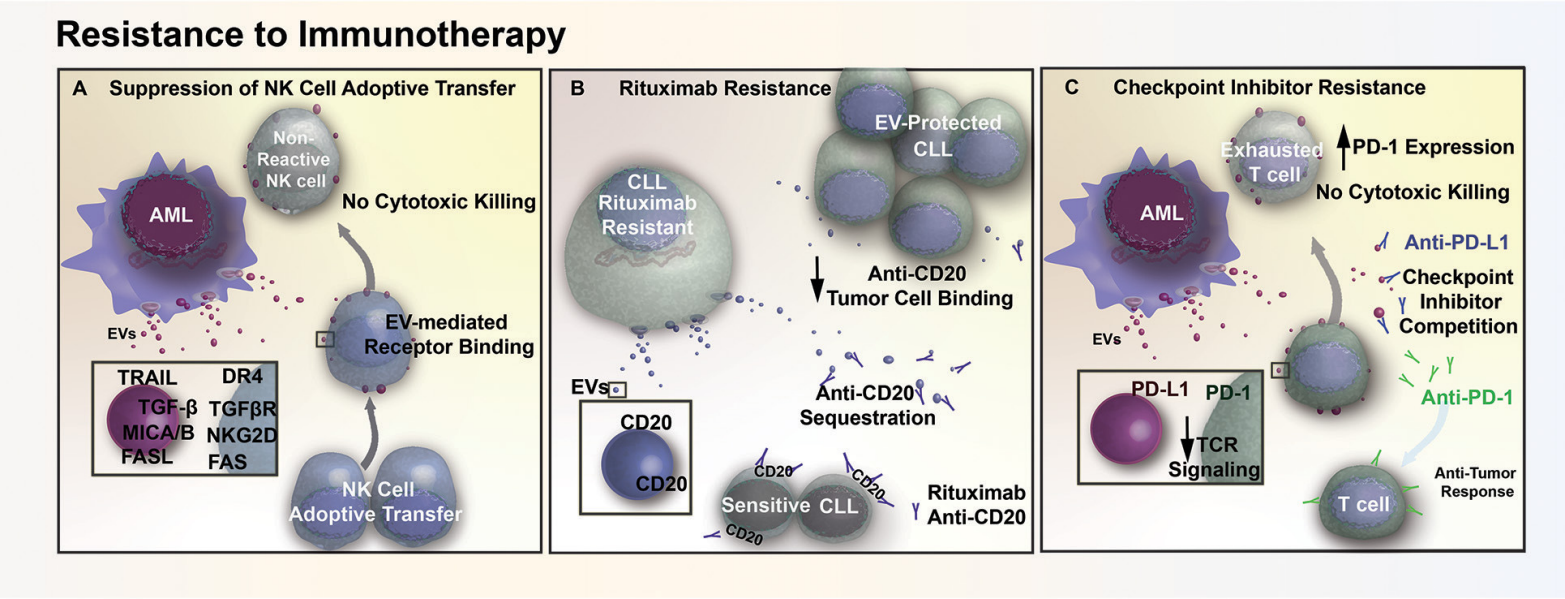
EV mediated resistance to immunotherapy. **(A)** AML EVs contain numerous immunosuppressive ligands (TRAIL, FASL, MICA/B) that reduce natural killer (NK) cell reactivity through receptor mediated binding. This EV-mediated signaling interferes with cell-based therapy, diminishing cytotoxic killing of tumor cells following adoptive transfer of NK cells. **(B)** EVs in CLL contain surface CD20, which acts as a decoy by sequestering Rituximab (anti-CD20) and preventing therapeutic antibodies from binding and opsonizing the tumor cells. **(C)** AML cells release EVs that contain the immunosuppressive ligand PD-L1. The transfer of PD-L1 via EVs reduces T cell activation in response to TCR stimulus, while also acting as decoys that compete with checkpoint inhibitor binding and prevent therapeutic antibodies from reaching their intended target.

While several chemoresistance mechanisms in leukemia involve the direct delivery of critical molecules via exosomes, resistance can also arise through immune dysregulation. For example, exosomes can reduce the efficacy of adoptive natural killer (NK) cell therapy in AML patients through interaction with activated NK-92 cells (79). More specifically, exosomes appeared to reduce the efficacy of activated NK-92 by transporting inhibitory ligands to NK-92 surface receptors, as demonstrated through a co-incubation study that exosomes derived from AML patients with NK-92 cells resulted in a 40% reduction of NKG2D receptor expression on NK-92 cell surface. As NKG2D receptor is involved in initiating a cytotoxic and cytokine response against threats, and inhibition of this receptor results in a reduction in cytotoxicity of NK-92 cells against AML blasts (Figure 3A). Exosome delivery of TGF-β to NK-92 cells is believed to be in part responsible for the decrease in NKG2D through TGFβRI/II pathway activation (79). Conceptually, exosomes may also contribute toward immunotherapy resistance through binding of antibodies to their surface. One study suggested that in CLL, exosomes may lower the bioavailability of rituximab, a common immunomodulatory antibody that targets the CD20 epitope on B-cells. Exosomal binding of anti-CD20 reduces circulating levels of rituximab, which in turn protects lymphocytic leukemia cells from anti-CD20 mediated opsonization (Figure 3B) and may explain why a number of CLL patients develop resistance to rituximab treatment (80).

AML cells also release exosomes that contain a potent immunosuppressive protein, programmed death-receptor ligand 1 (PD-L1) (79). PD-L1 binding to its cognate receptor, programed death-receptor 1 (PD-1), in both leukemia and solid tumors are able to suppress T cell activation in response to T cell receptor stimulation (81, 82). Expression of PD-L1 by tumor cells prevents T cell- and NK cell-mediated immune recognition and clearance, which increases the number of T cells with an “exhausted” and unreactive phenotype. It has been shown in both prostate cancer and melanoma that exosome-bound PD-L1 contributes to T cell suppression *in vitro* and *in vivo*. Additionally, exosomal PD-L1 has been shown to act as a decoy, sequestering anti-PD-L1 checkpoint inhibitors and outcompeting the anti-PD-1 checkpoint blockade for binding sites on cytotoxic CD8+ T cells (83, 84). Similar to prostate and melanoma models, we have found that AML EVs suppress T cell activation and adsorb anti-PD-L1 antibodies, suggesting that EVs may also contribute to immune checkpoint inhibitor resistance in AML (Figure 3C) (Butler, unpublished).

## Conclusion and Perspective

Recent research has uncovered numerous mechanisms through which EVs modulate proliferation, migration, and survival of malignant cells. While studies have implicated several specific proteins, cytokines, mRNAs, and miRNAs in the EV-mediated transfer of chemoresistance, the full spectrum of EV cargo involved in extrinsic chemoresistance remains to be fully defined. Additionally, the mechanisms through which many of these transferred miRNAs and proteins influence cell survival during chemotherapy are not fully understood. Investigation into leukemic stem cells and their role in transferring functional resistance factors similarly remains a potential focus for future AML EV research. *In vivo* trafficking of EVs could also provide additional perspectives on how the chemoresistance phenotype is being transferred (85). While phenotypic changes have been observed due to the trafficking of miRNAs and other molecules via EVs, these EVs may additionally influence cells through novel ligand-receptor mediated mechanisms with EV surface molecules. Here, a recent study provides an elegant approach to EV surface protein profiling, and it would be valuable to investigate whether EVs exhibit preferential targeting based on surface epitopes, and how this may alter chemoresistance (86). Recent work suggesting EV involvement in metastatic dissemination and functional conversion of other tissues is instructive, and may hold insight into the spread of AML to extramedullary sites (87). It will be critical to improve our understanding of dose and potency of EVs needed to confer drug resistance and whether a certain tumor burden must first exist, before AML-derived EVs have a significant influence on the leukemic niche. With numerous pathways implicated in the development of extrinsic chemoresistance, selectively targeting EV biogenesis or uptake represents a potential way to prevent intracellular leukemia cell survival signaling. As a proof-of-concept toward EV-uptake inhibition as a therapeutic strategy, Ortiz et al. recently showed that tumor EV-mediated pre-metastatic niche conversion can be prevented pharmacologically via administration of an EV-uptake inhibitor (87). On the other hand, Rab27 alpha and -beta (Rab27a/b) have both been identified as a critical modulators of EV biogenesis and secretion (39, 88). Thus, blocking EV biogenesis via loss of Rab27a/b function (89) represents a potential therapeutic approach. The ultimate goal, of course, is not so much to suppress EV trafficking *per se*, but rather to exploit our understanding of EV biology to identify cellular targets to overcome chemoresistance and achieve sustained long-term remissions. As an example, we previously have identified the role of miR-155, highly abundant in AML-EV, in the suppression of residual hematopoiesis in the AML BM (90). That study also introduced a discovery approach for novel EV-miRNA targets utilizing of molecular (RNA-induced silencing complex trap) and STING database (bioinformatic methodologies). Such an approach enables a broader understanding of complex AML-EV-mediated signaling and novel candidate targets (90). From a disease standpoint, minimizing and reversing the functional adaptation of the BM into a leukemia reinforcing compartment provides untapped opportunities to improve treatment outcomes in AML patients.

## Author Contributions

JN and PK conceived, wrote, and edited the manuscript. JB designed figures and wrote and edited the manuscript. D-WC wrote and edited the manuscript.

### Conflict of Interest

The authors declare that the research was conducted in the absence of any commercial or financial relationships that could be construed as a potential conflict of interest.
